# Editorial: Mechanisms and complexities underlying the cancer cell immune evasion and its therapeutic implications

**DOI:** 10.3389/fimmu.2025.1747904

**Published:** 2025-11-25

**Authors:** Zhexu Chi, Tapas Patra, Akhileshwar Namani

**Affiliations:** 1 Center for Regeneration and Aging Medicine, The Fourth Affiliated Hospital of School of Medicine, and International School of Medicine, International Institutes of Medicine, Yiwu, Zhejiang, China; 2 Department of Molecular Oncology, Sri Shankara Cancer Hospital and Research Centre, Sri Shankara National Centre for Cancer Prevention and Research, Sri Shankara Cancer Foundation, Bangalore, India

**Keywords:** immunotherapy, immune evasion, tumor microenvironment, therapeutic targets, biomarkers

Cancer immune evasion represents a central barrier to effective antitumor immunity and remains one of the most challenging hallmarks of cancer biology. The Research Topic “Mechanisms and Complexities Underlying the Cancer Cell Immune Evasion and its Therapeutic Implications” brings together diverse contributions that elucidate the cellular, molecular, and microenvironmental determinants of immune escape across malignancies. The collected works highlight how tumors exploit immunoregulatory pathways, remodel local immune niches, and shape therapeutic responses. Together, these articles provide an integrated understanding of cancer-mediated immune suppression and propose translational strategies to counteract it.

A major theme emerging from this topic is the central role of immunosuppressive cell populations. Liu et al. detail how regulatory T cells, myeloid-derived suppressor cells, leukemia-associated macrophages, and regulatory B cells orchestrate a profoundly immunosuppressive milieu in acute myeloid leukemia. Their review underscores the importance of targeting cellular recruitment and suppressive signaling pathways to restore effective anti-leukemic immunity. Wang et al. identify cytosolic thiouridylase CTU2 as a pan-cancer biomarker that modulates immune infiltration, tumor immunogenicity, and immunotherapy response. Their multitier analysis suggests that tRNA modification systems represent an underexplored axis of immune regulation. Complementing this, Chen et al. provide high-resolution insights into the heterogeneous immune microenvironment of colorectal cancer–origin ovarian metastases. Their genomic analyses reveal highly variable neoantigen loads, immune-desert phenotypes, and distinct metastatic routes, illustrating how spatial and clonal evolution shapes immune interactions and patient outcomes.

Few articles address specific signaling mechanisms and immunomodulatory pathways. Guo et al. review the inhibitory immune checkpoint TIM-3 in myelodysplastic syndromes, emphasizing its dual roles in tumor cell regulation and immune remodeling. Similarly, Han et al. integrate CRISPR-based functional genomics with transcriptomics to identify MELK-driven pathways that govern tumor progression, mutation burden, and immune contexture in clear cell renal cell carcinoma.

Mahamed et al. describe how cancer-derived exosomes convey immunosuppressive cargo that alter multiple immune cell populations, while engineered immune-cell–derived exosomes may counteract these effects. Beyond molecular pathways, this review highlights emerging systemic regulators of immune escape. Chen et al. expand this perspective, illustrating how multimodal intercellular communication ranging from metabolic competition to extracellular vesicle exchange and stromal interactions collectively drives CD8^-^ T-cell dysfunction across solid tumors.

Additional contributions broaden the conceptual landscape. Zhu et al. map the global bibliometric trends in tumor immune escape research, identifying shifting hotspots from classical checkpoint biology to metabolic reprogramming, microbiome interactions, and AI-driven immunotherapy prediction. Kovaleva et al. challenge the classical dichotomy of macrophage biology by showing that cytotoxic M1 macrophages may paradoxically promote tumor progression through selection pressure. Yan et al. provide a systematic-review demonstrating that bispecific antibodies combined with chemotherapy significantly improve survival outcomes in solid tumors, highlighting the translational potential of multi-target immunomodulation.

Collectively, all the articles in this Research Topic illustrate that immune evasion is not governed by a single pathway but emerges through complex, dynamic interactions between cancer cells, immune effectors, stromal elements, extracellular vesicles, and metabolic networks. These studies emphasize the need for integrated therapeutic strategies that target multiple axes of immune suppression at cellular, molecular, spatial, and metabolic levels.

As immunotherapies continue to evolve, a deeper mechanistic understanding of immune escape will be essential for improving patient outcomes, predicting response, and designing effective combination strategies. We thank all authors and reviewers for their valuable contributions and hope this Research Topic inspires further exploration into the intricacies of cancer–immune interactions and their therapeutic exploitation.

